# Supplementary material for: Psychometric Properties of the Authoritarian Attitude Scale in a Sample of Chilean Adolescent Students
Source: Behav Sci (Basel). 2025 Jun 1;15(6):756. doi: 10.3390/bs15060756 (PMC12189117; doi:10.3390/bs15060756)
Supplement: Supplementary file 1 [file behavsci-15-00756-s001.zip › behavsci-3578533-supplementary.pdf]

Material suplementario/ Supplementary Material

Escala Actitud Autoritaria, utilizada en el estudio actual/ Authoritarian Attitude Scale, used in the current study

Instrucciones/ **Instructions**:

A continuación, encontrarás cinco afirmaciones con las que puedes estar de acuerdo o en desacuerdo. Indica tu grado de acuerdo con cada afirmación/ **Below, you will find five statements with which you may agree or disagree. Please indicate your level of agreement with each statement.**

Versión original en español / Original version in Spanish

		Muy en desacuerdo	En desacuerdo	Algo de acuerdo	De acuerdo	Muy de acuerdo
1	Está justificado agredir a alguien que te ha quitado algo tuyo.	☐	☐	☐	☐	☐
2	Una relación de pareja es mejor si la mujer es sumisa.	☐	☐	☐	☐	☐
3	Un hombre debe hacer saber quién manda en casa.	☐	☐	☐	☐	☐
4	Para que la pareja funcione es mejor que el hombre gane más.	☐	☐	☐	☐	☐
5	Las mujeres son más débiles que los hombres.	☐	☐	☐	☐	☐
6	Está justificado pegar a alguien cuando te ha ofendido.	☐	☐	☐	☐	☐
7	Los inmigrantes deberían volver a su país tras un tiempo.	☐	☐	☐	☐	☐
8	Los inmigrantes son una amenaza para el futuro.	☐	☐	☐	☐	☐
9	La inmigración te preocupa.	☐	☐	☐	☐	☐
10	La violencia forma parte de la naturaleza de las personas.	☐	☐	☐	☐	☐

11	Pelearse de vez en cuando es normal.	☐	☐	☐	☐	☐

Versión en inglés / English version

		Strongly disagree	Disagree.	Somewhat agree.	Agree.	Strongly agree.
1	It is justified to attack someone who has taken something from you.	☐	☐	☐	☐	☐
2	A romantic relationship is better if the woman is submissive.	☐	☐	☐	☐	☐
3	A man should make it clear who is in charge at home.	☐	☐	☐	☐	☐
4	For a relationship to work, it's better if the man earns more.	☐	☐	☐	☐	☐
5	Women are weaker than men.	☐	☐	☐	☐	☐
6	It is justified to hit someone when they have offended you.	☐	☐	☐	☐	☐
7	Immigrants should go back to their country after a certain period of time.	☐	☐	☐	☐	☐
8	Immigrants are a threat to the future.	☐	☐	☐	☐	☐
9	La inmigración te preocupa	☐	☐	☐	☐	☐
10	Violence is part of human nature.	☐	☐	☐	☐	☐
11	Fighting from time to time is normal.	☐	☐	☐	☐	☐
